# Comparison of assembly process and co-occurrence pattern between planktonic and benthic microbial communities in the Bohai Sea

**DOI:** 10.3389/fmicb.2022.1003623

**Published:** 2022-09-29

**Authors:** Jinmei Liu, Xiaolei Wang, Jiao Liu, Xiaoyue Liu, Xiao-Hua Zhang, Jiwen Liu

**Affiliations:** ^1^Frontiers Science Center for Deep Ocean Multispheres and Earth System and College of Marine Life Sciences, Ocean University of China, Qingdao, China; ^2^Laboratory for Marine Ecology and Environmental Science, Qingdao National Laboratory for Marine Science and Technology, Qingdao, China; ^3^Institute of Evolution and Marine Biodiversity, Ocean University of China, Qingdao, China

**Keywords:** microbial community, community assembly, co-occurrence pattern, habitat differentiation, Bohai Sea

## Abstract

Unraveling the mechanisms structuring microbial community is a central goal in microbial ecology, but a detailed understanding of how community assembly processes relate to living habitats is still lacking. Here, *via* 16S rRNA gene amplicon sequencing, we investigated the assembly process of microbial communities in different habitats [water *verse* sediment, free-living (FL) *verse* particle-associated (PA)] and their impacts on the inter-taxa association patterns in the coastal Bohai Sea, China. The results showed clear differences in the composition and diversity of microbial communities among habitats, with greater dissimilarities between water column and sediment than between FL and PA communities. The microbial community assembly was dominated by dispersal limitation, ecological drift, and homogeneous selection, but their relative importance varied in different habitats. The planktonic communities were mainly shaped by dispersal limitation and ecological drift, whereas homogeneous selection played a more important role in structuring the benthic communities. Furthermore, the assembly mechanisms differed between FL and PA communities, especially in the bottom water with a greater effect of ecological drift and dispersal limitation on the FL and PA fractions, respectively. Linking assembly process to co-occurrence pattern showed that the relative contribution of deterministic processes (mainly homogeneous selection) increased under closer co-occurrence relationships. By contrast, stochastic processes exerted a higher effect when there were less inter-taxa connections. Overall, our findings demonstrate contrasting ecological processes underpinning microbial community distribution in different habitats including different lifestyles, which indicate complex microbial dynamic patterns in coastal systems with high anthropogenic perturbations.

## Introduction

Microorganisms are highly diverse in taxonomic composition and metabolic capability, and play important roles in global biogeochemical cycles (Prosser et al., 2007; Falkowski et al., 2008). Intensive efforts have been devoted on the microbial diversity and environmental associations, yet exploring the processes of community assembly is lacking but is crucial for a predictive understanding of their ecosystem functioning (Nemergut et al., 2013; Zhou and Ning, 2017). Deterministic and stochastic processes are two important mechanisms in structuring microbial community assembly (Zhou and Ning, 2017; Liu et al., 2019). On the basis of the niche theory, determinism is developed involving influence of biotic and abiotic factors, which are mainly reflected by species interaction and environmental filtering, respectively (Chesson, 2000). Stochasticity is based on the neutral theory assuming that all species are ecologically equivalent, and a community is controlled by processes such as birth, death, dispersal, and species formation (Chave, 2004). These two processes have distinct effects on microbial communities. Deterministic processes affect the fitness of microbial communities and determine composition and abundance (Li et al., 2019), whereas stochastic processes lead to unpredictable community changes (Tilman, 2004). There is an increasing effort in examining the relative contribution of deterministic and stochastic processes to microbial community assembly in different environments, such as soils (Jiao et al., 2020), freshwater (Mo et al., 2018), hot springs (He et al., 2021), and marine environments (Liu et al., 2020). However, no clear consensus has been obtained (Stegen et al., 2012; Wang et al., 2013; Dini-Andreote et al., 2015). It is considered that geographical scale and environmental gradients control the relative contribution of deterministic and stochastic processes (Hanson et al., 2012; Zhang et al., 2018). Changes in spatial scale can lead to differences in chemical gradients that may have implications for the ecological processes structuring microbial distribution patterns. For example, environmental factors (deterministic processes) including temperature, salinity, and sand size exerted a stronger influence than spatial factors (stochastic processes) on the benthic microeukaryotic communities in marine sandy beaches (up to 12,000 km; Zhang et al., 2018), whereas stochastic processes were the most important process in the assembly of archaeal communities in coastal sediments from the eastern Chinese marginal seas (up to 1,500 km; Liu et al., 2020). These studies suggest that the assembly of microbial community are not conserved among various ecosystems, emphasizing the importance for comparative analyses between environments (Allen et al., 2020; Li et al., 2021a).

The marine environment provides a variety of habitats, such as waters and sediments, which promote divergent adaption by adopting different lifestyles. Regarding water columns, two types of lifestyles exist, namely free-living (FL) and particle-associated (PA). The microbial communities inhabiting these habitats are distinctly different, and are likely assembled under different mechanisms (Wang et al., 2020; Gweon et al., 2021; Li et al., 2022). For example, Wang et al. (2020) found that homogeneous selection had a relatively higher importance in shaping bacterioplankton in the FL fraction, while this process was more important in structuring archaeaplankton of the PA fraction. By contrast, other studies reported that stochastic processes played a larger role in shaping both the FL and PA communities (Gweon et al., 2021; Shi et al., 2022). However, previous studies of marine microbial community assembly have mainly focused on a single habitat and few involve different habitats in a single study. Consequently, despite with an increasing effort devoted to explore the pattern of community assembly, a detailed understanding of how community assembly processes relate to living habitats is rare.

Examining the contribution of deterministic and stochastic processes in microbial community assembly can manifest the ecological strategies of coexisting species (Faust and Raes, 2012; Logares et al., 2020). Recently, correlation-based co-occurrence networks are frequently employed to describe microbial interactions, although they are controversial in reflecting true interactions (Liu et al., 2019). For instance, using the null model and co-occurrence networks, Li et al. (2021b) found that the dominant competition relationships between active bacterial and archaeal taxa could enhance the role of dispersal limitation in community assembly. By contrast, Mo et al. (2020) found that co-occurrence patterns of generalists and specialists were mainly driven by deterministic processes. Therefore, a complex network can reveal the inherent mechanism of microbial interactions after environmental changes and provide system-level insights for community assembly (Berry and Widder, 2014; Banerjee et al., 2018; Lv et al., 2019).

Our analyses were performed in the Bohai Sea (BS), a relatively isolated coastal area in the Chinese coastline, with little exchange with other seas and a long seawater renewal time (1.68 years; Li et al., 2015). The increasing anthropogenic activities have caused serious ecological problems to the BS, such as eutrophication, algal blooms, and hypoxia in the bottom water (Song et al., 2016; Zhai et al., 2019; Zheng et al., 2020). Consistently, microbial communities in the BS have been influenced by hypoxia (Wu et al., 2022), river input (Wang et al., 2021), and heavy metals (Ming et al., 2021). However, the microbial assembly pattern in this highly disturbed ecosystem is largely unknown. Moreover, the BS is a good candidate to investigate the community assembly in the marine environment covering a small spatial scale. In this study, we aimed to investigate whether microbial communities in different habitats (water *verse* sediment; FL *verse* PA) are assembled under different ecological processes and whether patterns of community assembly are related to species co-occurrences.

## Materials and methods

### Samples collection and environmental parameters

**Figure 1:**
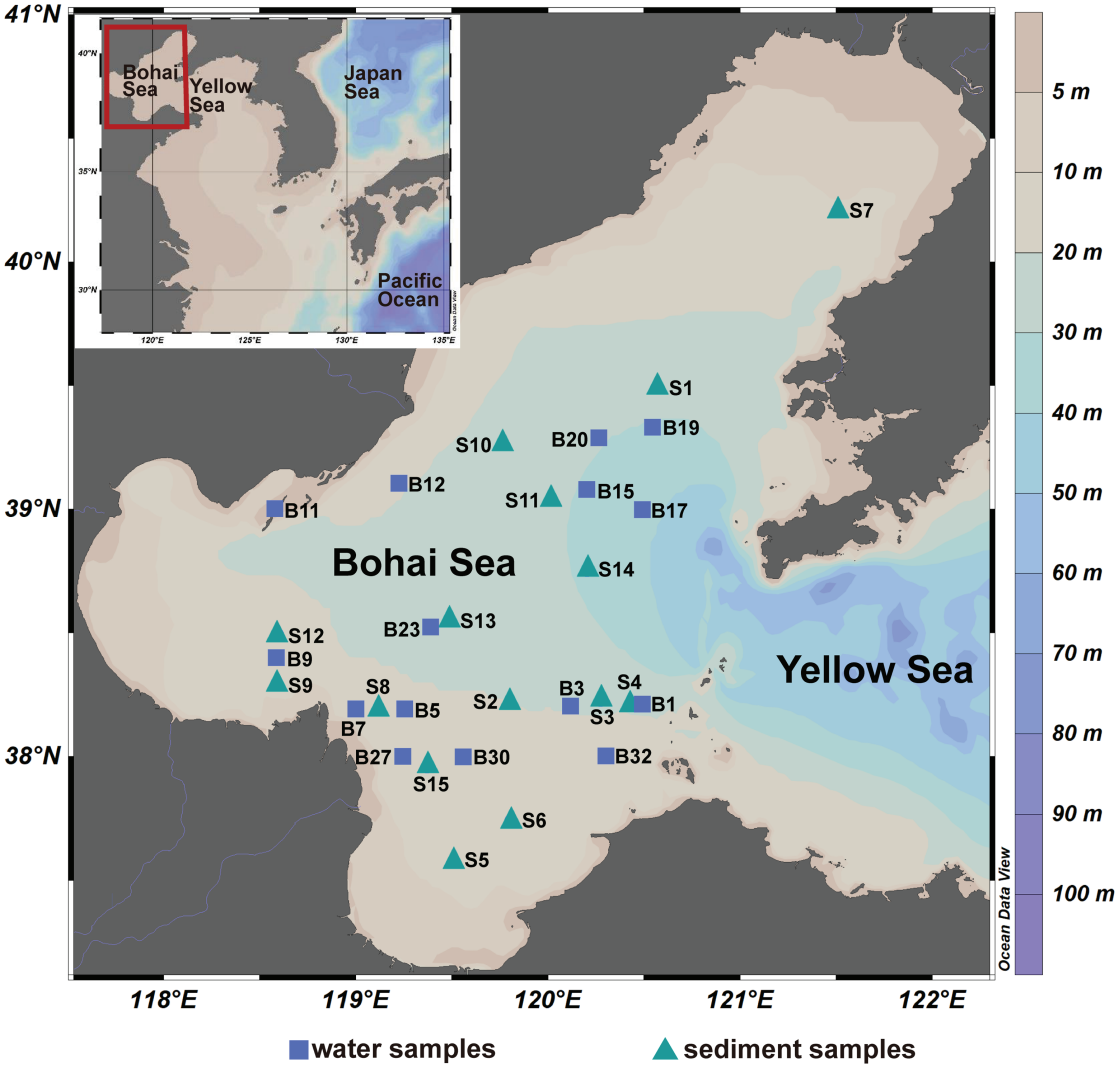
The sampling sites of the Bohai Sea, China.

Seawater and surface sediment samples were collected from the Bohai Sea (37°07′~40°56′ N, 117°33′~122°08′ E) of China during July–August 2019, and the location of sites are shown in Figure 1. Surface and bottom seawater samples were collected at 15 sites and were firstly filtered by a 3-μm filter to collect PA communities, and then filtered by a 0.22-μm filter to collect FL communities. The sediment samples (0–2 cm) were collected at 15 sites using a box corer and subsampled using a sterile spatula. The filter and sediment samples were stored at −20°C on board and at −80°C in the laboratory before DNA extraction. A total of 75 samples were finally obtained and divided into five groups according to the lifestyle of microbial community, namely Sur_FL (FL community in surface water), Sur_PA (PA community in surface water), Bot_FL (FL community in bottom water), Bot_PA (PA community in bottom water), and sediment. Salinity (26.6–32.1 PSU), temperature (17.8–28.5°C), pressure (2.9–41.3 dbar), pH (7.33–8.27), dissolved oxygen (3.57–8.96 mg/l), density (16.4–23.0 kg/m^3^), and fluorescence (0.29–7.82 mg/m^3^) of the seawater samples (surface and bottom waters) were determined using a Sealogger conductivity-temperature-depth (SBE 25, Sea-Bird Co.). The dissolved inorganic nutrients (nitrate, nitrite, ammonia, phosphate, and silicate) were analyzed by an auto-analyzer (SKALAR, sa3000/5000 chemistry unit).

### DNA extraction and sequencing

Total DNA was extracted from the seawater and sediment samples using the DNeasy PowerSoil Kit (QIAGEN, Germany) according to the manufacturer’s protocols. The primer pair 515F/806R (Walters et al., 2016) was used to amply the hypervariable V4 region of the 16S rRNA gene for bacteria and archaea. PCRs were conducted in a 20 μl reaction system, containing 0.4 μl of *TransStart* FastPfu polymerase, 2 μl of 2.5 mM dNTPs, 0.8 μl of 5 μM each primer, 4 μl of *TransStart* 5× FastPfu buffer, and 10 ng of template DNA. The PCR amplification conditions were as follows: 95°C for 3 min, followed by 35 cycles at 95°C for 30 s, 55°C for 30 s, 72°C for 45 s, and a final extension at 72°C for 10 min. The amplified PCR products were extracted from 2% agarose gel and purified using the AxyPrep DNA Gel Extraction Kit (Axygen Biosciences, Union City, CA, United States). The purified amplicons were sequenced on the Illumina Miseq PE300 platform (Illumina, San Diego, United States), performed by Majorbio Bio-Pharm Technology Co., Ltd., Shanghai, China. The raw sequencing data had been deposited in National Center for Biotechnology Information (NCBI) Sequence Read Archive (SRA) database under accession number PRJNA855144, and in the NODE database under accession number OEP003571.

### Sequencing data processing

The raw reads were firstly quality-filtered by fastp version 0.20.0 (Chen et al., 2018) to discard those of low average quality (<20) and short length (<50 bp), with any mismatch to barcodes and a maximum of two mismatches to primers. The high-quality reads were then merged by FLASH version 1.2.7 (Magoč and Salzberg, 2011). The merged reads were clustered into operational taxonomic units (OTUs) at a 3% dissimilarity level using UPARSE (Edgar, 2013). To reduce the potential of PCR bias, singleton OTUs were removed for downstream analyses. Taxonomic information for each OTU was assigned against the SILVA database (version 138)^1^ using the Ribosomal Database Project (RDP) classifier version 2.2^2^ (Wang et al., 2007) with a confidence threshold of 0.7. Chloroplast and mitochondrial 16S rRNA genes were removed from further analyses.

### Statistical analyses

All analyses were performed in the R software version 4.13 unless otherwise indicated.

To evaluate the diversity of microbial communities, alpha and beta diversity indices were calculated using the “vegan” package (Jari Oksanen et al., 2020). Comparison of the alpha diversity indices including Chao1, Shannon, ACE, and observed species was analyzed using the Kruskal–Wallis test. The beta diversity was estimated by Bray–Curtis distances. The community dissimilarity among different habitats was visualized by the non-metric multidimensional scaling analysis (NMDS). Statistical significance between groups was tested by permutational multivariate analysis (PERMANOVA) and analysis of similarities (ANOSIM). In order to evaluate the resources available to different microbial communities, we calculated the community-level niche breadth by the “niche.width “function of the “spaa” package (Zhang, 2016a), which is the average of niche breadth of a community (Wu et al., 2018).

The relationship between microbial communities and environmental factors was revealed by a Mantel test based on Pearson’s correlations. A dissimilarity matrix of community composition was calculated based on Bray–Curtis distances, which was carried out by the “linkET” package (Huang, 2021). A null model analysis was carried out using the statistical framework with 999 randomizations by Stegen et al. (2013) to quantify the relative contribution of deterministic and stochastic processes, namely homogeneous selection, heterogeneous selection, homogeneous dispersal, dispersal limitation, and drift. The beta nearest taxon index (βNTI) and the Bray–Curtis-based Raup-Crick metric (RC_bray_) are the two most important parameters of the framework to distinguish different ecological processes, calculated using the “picante” package (Kembel et al., 2010). The |βNTI| values higher than 2 indicate that community structure is mainly influenced by deterministic processes, where the βNTI values less than −2 or higher than 2 represent homogeneous and heterogeneous selection, respectively. The |βNTI| values falling within the range of −2 to 2 indicate that stochastic processes dominate the community assembly, and the specific stochastic processes can be further inferred by combining the RC_bray_ values. For |βNTI| < 2, RC_bray_ < −0.95, RC_bray_ > 0.95, and |RC_bray_| < 0.95 represent homogenizing dispersal and dispersal limitation and ecological drift, respectively. The neutral community model (NCM; Sloan et al., 2006) was further used to evaluate the potential roles of neutral processes in the assembly process by predicting the relationship between OTU relative abundance and their occurrence frequency. R^2^ is used as the coefficient of determination to assess the goodness of fit to the NCM, with higher values indicating that community assembly is more influenced by stochastic processes. The estimated migration rate (m) represents the probability of stochastic loss of OTUs in the community being replaced by dispersal from the metacommunity, with smaller values indicating that the community is more affected by dispersal limitation. The values of R^2^ and m were calculated by the “MicEco” package (Russel, 2021).

Meta-community co-occurrence network based on Spearman correlation coefficients was constructed using the “igraph” (Csardi and Nepusz, 2006) and “psych” (Revelle, 2021) packages, and visualized using Gephi version 0.9.2.^3^ To reduce the complexity of the network for a better visualization, only the top 500 OTUs were used for network construction. The pairwise Spearman’s rank correlations were calculated to visualize the associations between species (at OTU level), with correlation coefficients |r| > 0.7 and false discovery rate-corrected *p* value < 0.01 being considered as a valid relationship. The topological features of the network including network level (average degree, clustering coefficient, average path length, modularity, graph density, network diameter, betweenness centralization, and degree centralization) and node level (node degree, node transitivity, betweenness centrality, and closeness centrality) were calculated. The subnetwork of different communities was extracted from the meta-community network.

## Results

### Composition of microbial communities

**Figure 2:**
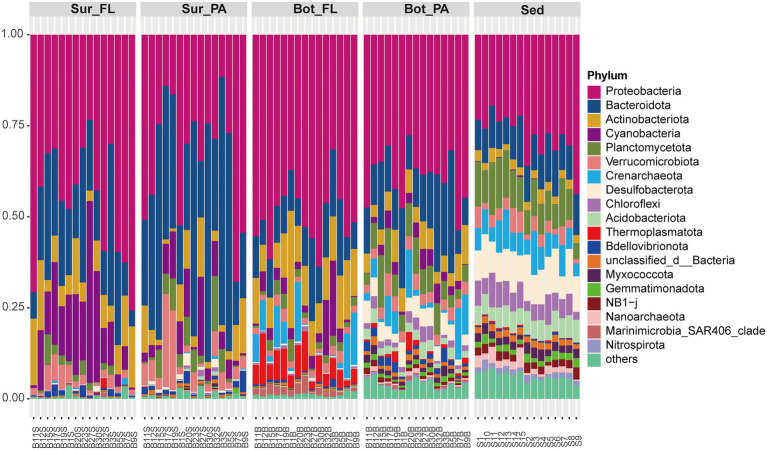
Composition of microbial communities at the phylum level. Sur_FL, FL community in surface water; Sur_PA, PA community in surface water; Bot_FL, FL community in bottom water; Bot_PA, PA community in bottom water; Sed, sedimentary community.

Illumina sequencing generated a total of 1,373,925 clean reads, which were clustered into 15,846 OTUs at a 97% sequence similarity level. The flattening of the species accumulation curve indicated that the sequencing data were able to recover most of the local species (Supplementary Figure 1). The sequences were taxonomically diverse and affiliated with a total of 78 phyla. The dominant phyla were *Proteobacteria* (39.4%), *Bacteroidota* (19.1%), *Actinobacteriota* (7.3%), and *Cyanobacteria* (6.0%), which accounted for more than half of the community (Figure 2). These four phyla varied considerably between water depths and between water column and sediment. They showed a decreasing trend from surface water (88.0%), bottom water (71.0%) to sediment (40.8%). As expected, the abundance of *Cyanobacteria* especially *Synechococcus* peaked in surface seawater (11.1%) and decreased in bottom water (3.8%) and sediment (0.1%; Figure 2; Supplementary Figure 2). By contrast, archaeal phyla *Crenarchaeota* and *Thermoplasmatota*, mainly represented by *Nitrosopumilaceae* and Marine Group II, respectively, were more abundant in bottom water than in surface water (Kruskal–Wallis test, *p* < 0.001). Compared to seawater, the sediment samples were relatively enriched with *Planctomycetota*, *Desulfobacterota*, *Chloroflexi*, *Acidobacteriota,* and *Nitrospirota* (Kruskal–Wallis test, *p* < 0.001). Abundant orders in the sediment samples included *Anaerolineales* (*Chloroflexi*), *Thermoanaerobaculales* (*Acidobacteriota*), *Desulfobacterales* (*Desulfobacterota*), and MSBL9 (*Planctomycetota*).

**Figure 3:**
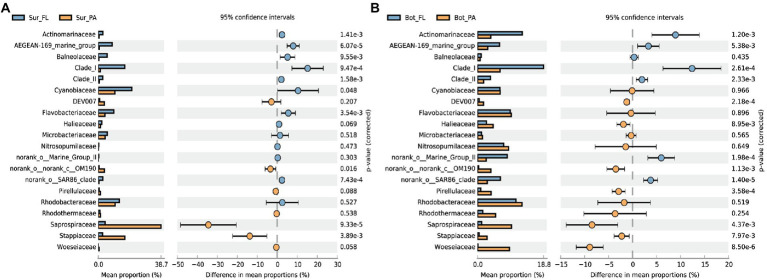
Differences in community composition between lifestyles at the family level.

Significant compositional changes were also observed between the FL and PA communities (Figure 3; Supplementary Figure 3). The lifestyle-based community variance appeared to be more evident in bottom water than in surface water, with more phyla being enriched in PA than FL (Supplementary Figure 3). At a finer taxonomical level, *Actinomarinaceae*, Clade I (SAR11), and AEGEAN-169 marine group were more abundant in the FL fraction, whereas the PA community especially in surface water was relatively enriched by *Saprospiraceae* belonging to *Chitinophagales* in *Bacteroidota*, followed by *Stappiaceae* (Figure 3A). In the bottom water, more microbial families were enriched in PA than in FL fractions, and the PA-enriched families were also dominant in the sediment, such as *Woeseiaceae*, *Pirellulaceae*, and *Halieaceae* (Figure 3B).

### Diversity of microbial communities

**Figure 4:**
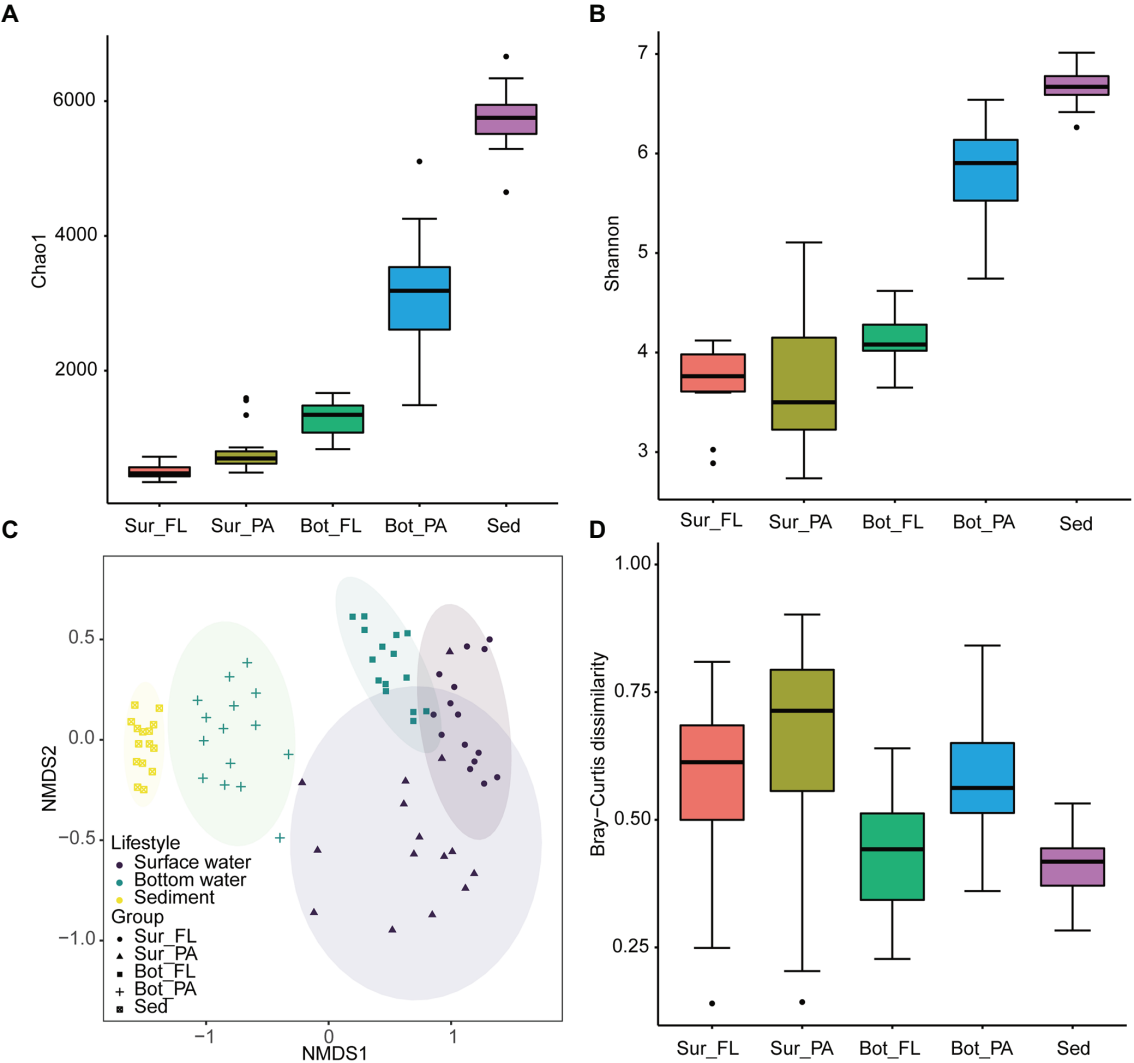
Alpha and beta diversity of microbial communities at the OTU level. **(A)** Chao1 index of different communities; **(B)** Shannon index of different communities; **(C)** NMDS plot of different communities; **(D)** Beta diversity index of different communities.

ACE, observed species, Chao 1, and Shannon indices exhibited similar variation trends, with the highest diversity level found in sediment, followed by bottom water and surface water (Kruskal–Wallis test, *p* < 0.001; Figures 4A,B; Supplementary Figures 4A,B). In bottom water, the alpha diversity indices were significantly higher in the PA than the FL fractions (*p* < 0.001). No similar trend was observed in the surface water, where the average value of Shannon index appeared to be higher in FL than in PA communities though with no statistical significance (Figure 4B).

NMDS analysis based on the Bray–Curtis distance showed that the microbial communities can be grouped into five clusters, i.e., Sur_FL, Sur_PA, Bot_FL, Bot_PA, sediment (Figure 4C). The pairwise PERMANOVA analysis further supported this clustering pattern and showed that the microbial community was more variable between habitats (sediment *verse* water) than between lifestyles (FL *verse* PA; Supplementary Table 1). Although separated from seawater samples, the sedimentary community showed a high similarity to the Bot_PA community. The Sur_FL community was more similar to the Bot_FL than to the Sur_PA community (Supplementary Table 2; Supplementary Figure 5), which indicated that niche partitioning by lifestyle was more evident than that by water depth. Furthermore, there was a greater level of intra-group variation for planktonic than benthic communities, and the PA communities exhibited higher heterogeneity compared to those in the FL fraction (Figure 4D).

### Niche breadth of microbial communities

**Figure 5:**
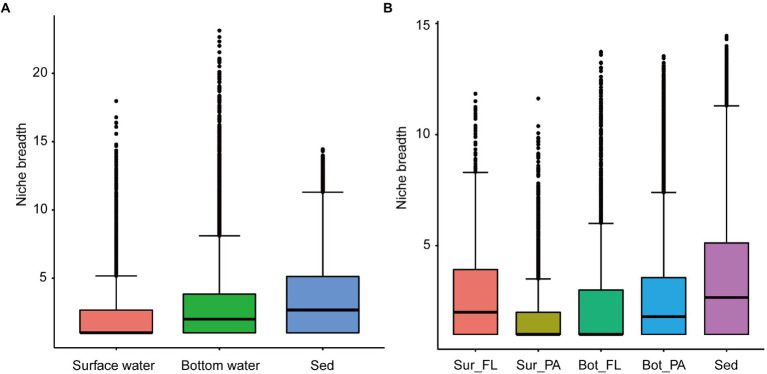
Niche breadth of microbial communities. **(A)** Comparison of habitat niche breadth of surface water, bottom water, and sediment; **(B)** Comparison of habitat niche breadth of Sur_FL, Sur_PA, Bot_FL, Bot_PA, and sediment.

The community-level niche breadth values varied significantly among habitats (Kruskal–Wallis test, *p* < 0.001; Figure 5). The niche breadth was highest in sediment, moderate in bottom water, and narrowest in surface water (Figure 5A). In surface water, the niche breadth values were significantly higher for the FL community than for the PA community (Figure 5B; *p* < 0.001); however, an opposite trend was observed for the niche breadth values in bottom water (Figure 5B; *p* < 0.001).

### Correlations of microbial communities with environmental factors

**Figure 6:**
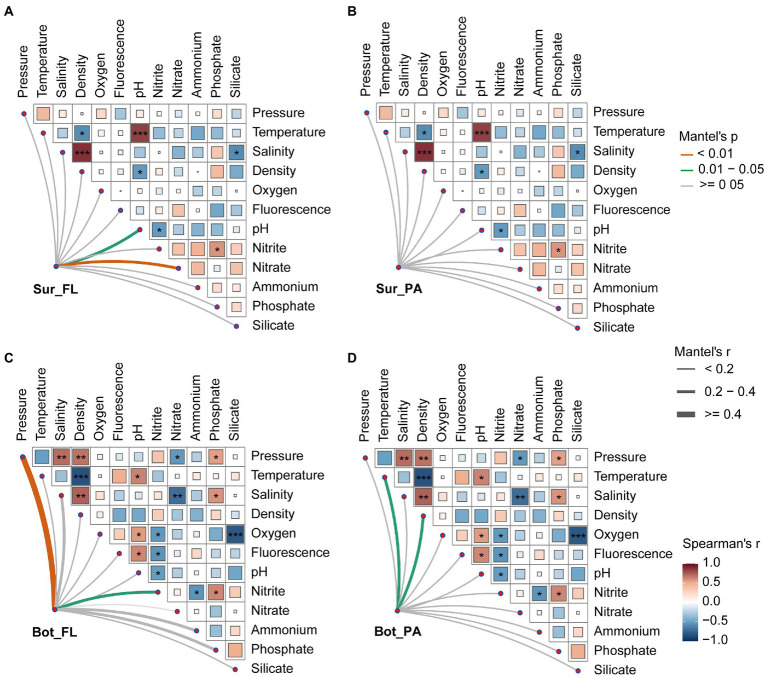
Relationships between microbial community and environmental factors in surface and bottom waters. **(A)** Sur_FL community; **(B)** Sur_PA community; **(C)** Bot_FL community; **(D)** Bot_PA community. Pairwise comparisons of environmental factors are shown, with a color gradient denoting Spearman’s correlation coefficients.

To further investigate the underlying mechanisms that structure the microbial community in different habitats, partial Mantel test was performed to correlate distance-corrected dissimilarities of microbial community with those of environmental factors (Figure 6). A detailed description of the physical and chemical properties in surface and bottom waters is shown in Supplementary Table 3. A variety of environmental factors showed correlations with the different microbial communities. In surface water, the FL community was positively correlated with nitrate (*r* = 0.40, *p* < 0.01) and pH (*r* = 0.23, *p* < 0.05), whereas the PA community showed no significant correlations with environmental factors. In bottom water, pressure (*r* = 0.52, *p* < 0.01), and nitrite (*r* = 0.26, *p* < 0.05) correlated to the FL community, while temperature (*r* = 0.38, *p* < 0.05) and density (*r* = 0.28, *p* < 0.05) correlated to the PA community.

### Assembly process of microbial communities

**Figure 7:**
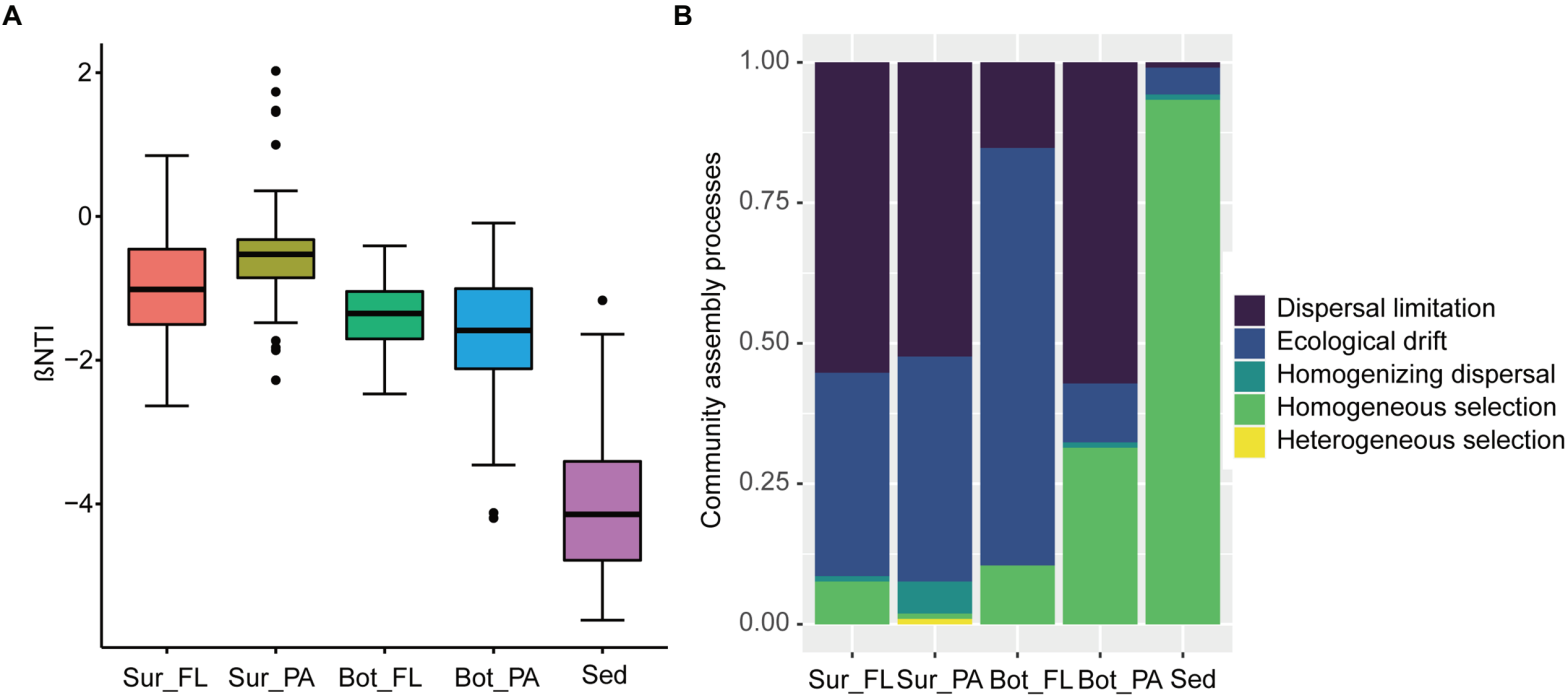
Community assembly mechanisms in different communities. **(A)** βNTI values for different communities; **(B)** The assembly processes of different communities with the null model.

The |βNTI| values showed that FL and PA communities in surface and bottom waters were mainly influenced by stochastic factors, whereas the effect of deterministic processes was more important than stochastic processes in the benthic communities (Figure 7A). Dispersal limitation and ecological drift of stochastic processes dominated the assembly of microbial communities in surface and bottom waters, but homogeneous selection of deterministic processes was overwhelmingly dominant in the sedimentary community with a proportion of 93.3%. Similarly, the relative contributions of ecological processes to the assembly of communities in different habitats were distinct (Figure 7B). For example, dispersal limitation exerted a great role in Sur_FL (55.2%), Sur_PA (52.4%), and Bot_PA (57.1%) communities, whereas ecological drift contributed a large fraction to the community assembly in Bot_FL (74.3%). Moreover, homogeneous selection had a higher effect on communities in bottom water (10.5% for Bot_FL and 31.4% for Bot_PA) than in surface water (7.6% for Sur_FL and 0.95% for Sur_PA). Homogenizing dispersal and heterogeneous selection played minor roles in the communities.

The NCM was also performed to verify the results from the null model. The NCM fitted well to the community assembly, showing that stochasticity played an unneglectable role in the assembly of the microbial communities. The degree of fit (R^2^) was higher in seawater than in sediment, but the migration rates (m) exhibited an opposite trend (Supplementary Table 4), which were in line with the null model analysis. In general, both deterministic and stochastic processes affected the microbial community assembly, and their relative contribution varied across microbial communities in different habitats.

### Co-occurrence pattern of microbial communities

**Figure 8:**
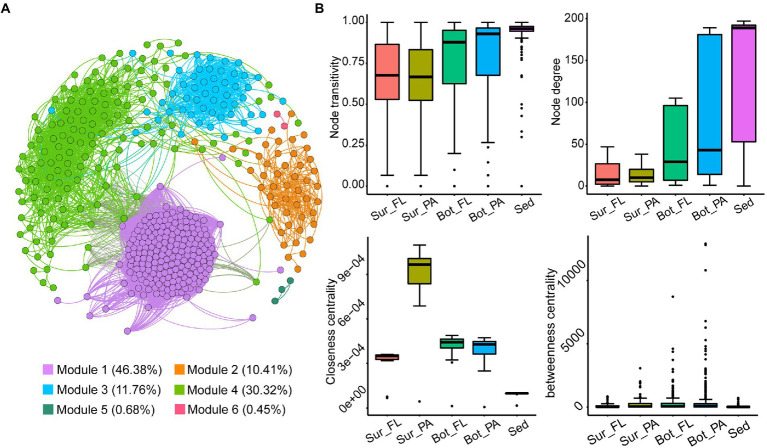
Co-occurrence networks of the microbial community in all samples and topological properties. **(A)** OTU level co-occurrence network; **(B)** Comparison of node-level topological features among subcommunities.

A co-occurrence network of 442 nodes and 21,188 edges was generated for the microbial community based on Spearman’s correlation coefficients (Figure 8A). The network consisted of 6 modules, of which modules 1, 2, 3, and 4 accounted for 46.4%, 10.4%, 11.8%, and 30.3% of the whole network, respectively (Figure 8A). The nodes in the network were assigned to 37 identified phyla, accounting for 85.3% of all nodes. *Nanoarchaeota* (22.9%), *Planctomycetota* (10.6%), *Patescibacteria* (10.0%), *Proteobacteria* (7.0%), *Verrucomicrobiota* (4.8%), *Chloroflexi* (4.5%), and *Bacteroidota* (3.4%) mainly occupied the nodes. The majority of edges (98.0%) in the network were positive, indicating a predominantly cooperative relationship across the microbial community.

To estimate the extent of species co-existence in different habitats, network topology parameters for each of the community groups were calculated (Figure 8B; Supplementary Table 5). The network of different communities demonstrated distinct co-occurrence patterns. Comparison of the network topological parameters showed the complexity of the networks increased from seawaters (surface and bottom waters) to sediment. Specifically, the sediment network had higher clustering coefficient, average degree and graph density, and lower average path length and network diameter than the networks in seawater, suggesting that the benthic community was more interconnected. In seawater, higher clustering coefficient, average degree, and graph density were observed in the bottom water than in surface water. PA communities consistently exhibited much closer interconnections than FL communities, which was confirmed by higher values of the topological parameters. In addition, the node-level topology showed an increased trend in node degree and node transitivity from the surface water to the sediment, and the lowest closeness centrality and betweenness centrality were observed in the sediment network.

## Discussion

Exploring the mechanisms that govern community assembly has been a central task in microbial ecology. In this study, we examined the assembly mechanism of FL and PA communities in seawater of the BS and compared it with that of the sediment populations. We found that the dissimilarities in community between water column and sediment were greater than those between FL and PA communities. Dispersal limitation, homogeneous selection, and ecological drift were the dominant processes in microbial community assembly but showed obvious difference among habitats. Our results suggest that ecological processes underlying the microbial community reflect the influence of environmental homogeneity and the potential influence of human disturbance, niche selection, and putative interspecies interactions.

### Varying compositions of microbial community in different habitats

In line with some studies in the BS (Chen et al., 2020; Guo et al., 2022; Wu et al., 2022), *Proteobacteria*, *Bacteroidetes*, and *Cyanobacteria* were found to be dominant groups and were abundant in both water and sediments. These groups have been characterized as the important groups in eutrophic coastal ecosystems (Klawonn et al., 2016; Gomes et al., 2019; Zhang et al., 2019; Zhao et al., 2021). However, clear differences in the relative abundance of several dominant microbial phyla were seen between water and sediment samples. *Cyanobacteria* especially *Synechococcus* and the proteobacterial SAR11 clade were abundant in the water samples, supporting the global importance of *Synechococcus* as a clade of primary producer, especially at coastal sites (Nagarkar et al., 2021), and the cosmopolitan nature of SAR11 in the global ocean (Giovannoni, 2017). The sedimentary communities were featured by higher abundances of *Desulfobacterota*, *Planctomycetota*, *Chloroflexi*, *Acidobacteriota*, and *Nitrospirota*, probably because they have enhanced capability in mineralization of organic matter and degradation of pollutants under anoxic conditions (Petro et al., 2017). For example, *Desulfobacterota* and *Acidobacteriota* are involved in the sulfate and/or sulfur-related processes (Chen et al., 2020) and nitrate reduction processes (Kielak et al., 2016), respectively. Previous studies have reported increased abundance of sulfate-reducing bacteria of *Desulfobacterota* in coastal than in open ocean sediments (Liu et al., 2015). The capability of *Desulfobacterota* in degrading organic pollutants (Ayangbenro et al., 2018) indicated that anthropogenic activities can lead to considerable organic pollutants in BS, which may affect the composition pattern of a microbial community.

FL and PA communities have been demonstrated to be compositionally different (Ortega-Retuerta et al., 2013; Bižić-Ionescu et al., 2014; Rieck et al., 2015; Roth Rosenberg et al., 2021), which was also the case in the BS. In addition to this information, we found that the size fractioned community dissimilarities were greater than those caused by depth. The indication was that there was a strong niche partition by lifestyle in the shallow basin, which may be created by river-transported particles and algal growth. This was reflected by a higher proportion of *Chitinophagales* (represented by an unclassified genus) in the PA community. *Chitinophagales* belonged to *Bacteroidetes* containing members associated with algal-derived organic matter degradation (Krüger et al., 2019). Indeed, members of *Chitinophagales* have been found to be positively correlated with algal biomass productivity (Steichen et al., 2020). Attachment to particles may also provide a shelter for microbes, facilitating escape from the influence of changing environments in seawater (Yung et al., 2016). This was consistent with our finding that the PA communities especially in the surface water were less affected by environmental factors compared to its FL counterpart. It was notable that the PA communities in the bottom water were more similar to the sedimentary communities with increased abundance of *Chloroflexi* and *Desulfobacterota* in favor of anoxic conditions. This was not expected since the bottom water samples did not reach hypoxia. Alternatively, we hypothesized that the sediment resuspension event (Wang et al., 2014) may bring the surface sediment-associated cells into the bottom water layers. The residence time and ecological effect of the sedimentary dwellers in the bottom water need further characterization.

Differences in community composition reflect habitat differentiation, which in turn affects the diversity of microbial communities. As expected, the diversity of microbial community in different habitats was distinct. According to the “more individuals hypothesis,” higher resource availability would increase the number of individuals and thus sustain higher microbial diversity (Storch et al., 2018; Furness et al., 2021). In our study, the microbial alpha diversity was higher in sediment than in waters. The sedimentary communities were developed under the effect of long-term sediment accumulation, deposition, and erosion. Therefore, microbial colonization and enrichment on nutrient-rich substrates may be an important reason for the high diversity of the sedimentary community. Similarly, particles can provide a nutrient-richer environment relative to the surrounding water (Azam and Malfatti, 2007), which may have resulted in a higher species diversity in the bottom water PA community (Bižić-Ionescu et al., 2014; Rieck et al., 2015; Yung et al., 2016). Alternatively, this more diverse PA community in bottom water may be contributed by resuspension of sediment-associated cells as noted before. Such a hypothesis was supported by the observation of a similar alpha diversity level between the FL and PA community in surface water. The over-representation of *Chitinophagales* may have resulted in the relatively less diverse surface PA community. In our study, the microbial community with high alpha diversity tended to have a wider niche breadth, whereas the community with a wider niche breadth tended to have lower beta diversity. As expected, the narrowest niche breadth was observed in the surface PA community, which implied that the number of available niches for the PA community to colonize was relatively limited. An increase in niche breadth would raise the number of taxa at a particular site and increase the probability of the same taxon being present at different sites, resulting in an increased alpha diversity and a decreased inter-site beta diversity (Okie et al., 2015). Additionally, different microbial taxa have distinct ecological niches, and only a subset of a regional collection of species can survive and reproduce under local conditions (Okie et al., 2015). Therefore, niche selection may be one of the reasons for the lower Shannon index in the surface PA community predominated by *Chitinophagales*.

### Ecological processes underlying the community assembly of seawater and sediment

Deterministic and stochastic processes are believed to jointly determine the assembly of microbial communities, but gaps remain with regard to their relative importance (Stegen et al., 2013; Dini-Andreote et al., 2015; Zhou and Ning, 2017; Vass et al., 2020). Contrary to the macroecological theory in which the relative impact of stochasticity increases with resource supply (Chase, 2010), the less effect of stochasticity in nutrient-rich sediments compared to seawater suggested a negative resource supply-stochasticity relationship. Such a relationship has also been observed in soil ecosystems, implying that the relationship between resource supply and stochasticity is dependent on the extent of physicochemical gradients that impose selection (Dini-Andreote et al., 2015). In addition to physicochemical conditions, stochasticity may also be affected by the interaction between dispersal and selection strength (Evans et al., 2016) as well as spatial scale (Martiny et al., 2011).

Previous studies have demonstrated that dispersal limitation played an important role in community assembly (Nemergut et al., 2013; Stegen et al., 2015; Liu et al., 2020). This ecological process was considered to be resulted from large spatial distance and/or steer environmental variance (Zhou and Ning, 2017). The dispersal capacity of microbes is generally thought to be higher in seawater than in sediment (Zinger et al., 2014; Liu et al., 2020). However, our results revealed a higher effect of dispersal limitation in seawater, especially in the bottom PA fraction. One plausible explanation for this is that the planktonic bacteria in BS seawater are largely affected by particles as noted earlier. This was similar to the previous report that dispersal limitation played a more important role in the assembly process of the bacterial community on microplastics than those in water and sediments (Zhang et al., 2022). Association with particles can decrease the chance of dispersal. Although particles may also spread in seawater, the abundant Chloroplast 16S rRNA gene sequences in the PA fraction (data not shown) reflected high algal biomass due to eutrophication. The potential large particle size of eukaryotic algae may have increased the possibility of dispersal limitation. Difference in environmental conditions and micro-niches provided by the particulate matter would act as a hindrance and make it more difficult for microbial dispersal (Okie et al., 2015). Alternatively, due to frequent human activity-derived disturbance, the planktonic microorganisms may have evolved to tolerate the environmental dynamics and thus respond weakly to deterministic factors (Liu et al., 2020). Across water depths, increased effect of dispersal limitation with water depth due to wind decay has been found in the assembly of bacterial community in the East China Sea (Wu et al., 2018). However, we showed here that dispersal limitation exerted a greater influence in the surface than the bottom FL community. The shallow water depth of BS causing similar wind effect across depths and the potential effect of algae in the surface water may act as possible explanations.

Low or limited dispersal can introduce stochasticity by increasing ecological drift (Hanson et al., 2012; Stegen et al., 2013; Dini-Andreote et al., 2015), whereas high dispersal rates result in higher microbial biomass and species diversity and can introduce mass effects (Evans et al., 2016). Consistently, in this study, stochasticity contributed by ecological drift was a vital aspect of community assembly in seawater, particularly in FL communities. And, our result was in line with the hypothesis that drift will become dominant under weak selection pressure and low species diversity (Chase and Myers, 2011). Indeed, the lower alpha diversity of FL relative to PA communities in bottom water leads to a stronger effect of ecological drift on the FL community assembly.

The sediment habitats are often more heterogeneous and have a lower regional connectivity, decreasing the probability of active dispersal in sedimentary microorganisms (Zinger et al., 2014). Thus, it was considered that sedimentary microorganisms may have a lower dispersal capacity, which may enhance the impact of dispersal limitation on community assembly (Östman et al., 2010). However, both the null model and neutral model showed that the sedimentary community experienced lower dispersal limitation compared to the seawater community. The sediments could have mobility due to transport by water currents (Liu et al., 2018), causing the sediment resuspension event facilitating cell dispersal. Another potential reason may be that microbial communities can be constrained by niche breadth. Niche breadth generally represents the range or variety of conditions defining a species’ niche and the adaptability of a species to its environment is defined by niche breadth (Sexton et al., 2017). On this basis, the degree of niche breadth may be determined by the microbial sensitivity/resistance in response to environmental gradients, thus reflecting the importance of environmental filtering (deterministic process). Indeed, previous studies have shown that species with wider niche breadths are influenced less by environmental factors (Pandit et al., 2009; Wu et al., 2018). In this study, microbial communities inhabiting sediments have significantly higher niche breadth than those in seawater, which may be determined by the small spatial scale-covered and low environmental variances. Thus, the influence of deterministic factors was expected to be low. However, homogeneous selection was the most important ecological process explaining more than 90% of the sedimentary community variation. The indication of homogeneous selection is that similarity in some sedimentary factors such as the high content of organic matter and pollutions (Gao et al., 2014; Zhao et al., 2018) may have led to habitat homogenization (Liu et al., 2020; Zhang et al., 2021) and select similar communities across samples, although environmental factors of the sediment samples were not measured here. Therefore, despite high deterministic contribution, the consistent selection pressures driven by consistent environmental conditions underpin the similarity of community composition in the sediment and lead to high niche breadth values. This selection minimizes the role of stochastic processes and drives similarity in local community structures (Evans et al., 2016).

### Linking community assembly processes and co-occurrence patterns: Homogeneous selection enhances the connectivity of microbial communities

Microorganisms occupying a specific ecological niche can form complex interaction networks (Faust and Raes, 2012). Hence, interspecific interactions are important factors structuring microbial community assembly (Yuan et al., 2019; Ortiz et al., 2021). Many studies have shown that diverse microbial groups can influence other taxonomic groups by interactions (Morriën et al., 2017; Chen et al., 2019; Trivedi et al., 2020). Co-occurrence networks have been frequently used to infer potential interactions, although topology-based system approach did not reflect true inter-taxa correlations (Liu et al., 2019). Nodes in the constructed network were comprised of many rare taxa such as *Nanoarchaeota* and *Patescibacteria.* This implied that rare species could play key roles in maintaining the microbial community structure, in line with previous studies in soil (Lupatini et al., 2014) and deep-sea sediments (Zhang et al., 2021). The majority of connections (>96%) in the networks were positive, likely indicative of more frequent putative cooperation than competition. On the other hand, the co-occurrence of many microorganisms may reflect cross-feeding, co-aggregation, co-colonization, and niche overlap (Faust and Raes, 2012; Faust et al., 2015; Shi et al., 2019). For example, *Bacteroidetes*, one of the dominant groups in samples of this study, can degrade polysaccharides (Gómez-Pereira et al., 2011) and their degradation products can promote the growth of other heterotrophic bacteria (Nagata et al., 2003). Additionally, the high-complexity networks tend to have greater stability in communities (Mougi and Kondoh, 2012). The relatively high community stability indicated by the more clustered network structures and stronger connections in sediments compared to seawater may be facilitated by efficient putative resource and information transfer between species (Morriën et al., 2017). Consistent with previous studies (Zhang et al., 2016b; Xu et al., 2018), the network of the PA communities in seawater was more complex than the FL communities, which indicated that PA communities exhibited higher stability than the FL communities. The reasons for this may be that PA communities are physically closer to their collaborators or competitors within the relatively confined particle space, thus facilitating putative metabolic cooperation and genetic exchange (Zhang et al., 2014, 2016b; Dang and Lovell, 2016). And, the large amount of nutrients on the particles could create more suitable micro-niches for the PA communities (Xu et al., 2018); these particulate micro-niches might play a vital role in shaping the structure of PA communities (Zhang et al., 2016b).

Determining the linkages between community assembly and species coexistence is essential to understand the mechanisms that maintain community diversity (Vályi et al., 2016; Jiao et al., 2020; Li et al. 2021a). By constructing the linkage between assembly processes and species co-occurrence in microbial communities, Jiao et al. (2020) found that the microbial co-occurrences tended to be higher when agricultural soil microbial communities were mainly driven by dispersal limitation. By contrast, Li et al. (2021a) found that deterministic processes (mainly heterogeneous selection) became a less dominant ecological process in the intermonsoon communities in the Eastern Indian Ocean as the co-occurrence increased. These linkages were thought to be largely related to environmental filtering (Jiao et al., 2020; Li et al., 2021a). In this study, an opposite trend was observed that stochastic processes become less dominant as the connection of microbial communities increased. The reason may be that the different mechanisms selected for microbial community assembly in different habitats, thus causing microbial communities in specific ecological niches to change their coexistence strategies. The contemporary coexistence theory highlights that coexistence depends on niche differences and fitness differences (HilleRisLambers et al., 2012). The wider community-level niche breadth observed in sediments would favor coexistence of different species by reducing direct competition, namely niche complementarity (Konopka et al., 2015). Compared to seawater, the sedimentary community had higher diversity, accompanied by the high positive correlation (99.3%) in the network. Therefore, microbial associations could contribute to microbial assembly by acting as a selective force to promote diversity.

## Conclusion

In this study, we compared the diversity, community composition, assembly processes, and co-occurrence patterns of microbial communities in different marine habitats in BS, including those between seawater and sediment and between FL and PA lifestyles. The most dominant taxa were *Proteobacteria*, *Bacteroidota*, *Actinobacteriota*, and *Cyanobacteria*. The community alpha diversity showed a gradual increase from surface water to sediment, which was accompanied with the decreased beta diversity and the increased average niche breadth values. Furthermore, we provided evidence that the community of different living habitats was structured by different ecological processes. In contrast to a higher contribution of stochastic processes in seawater community, the sedimentary community was mainly shaped by deterministic processes. Concomitant with a decrease in the contribution of stochastic processes from surface water to sediment, the degree of microbial associations increased. We also showed distinct assembly pattern between the FL and PA communities. The dynamics of microbial communities and assembly patterns may be contributed by the local environmental conditions, ocean currents, and to a larger extent by human activities. Overall, our results demonstrated habitat-specific community assembly and potential linkages with association patterns, underscoring the importance for an integrated understanding of microbial community dynamics.

## Data availability statement

The names of the repository/repositories and accession number(s) can be found at: https://www.ncbi.nlm.nih.gov PRJNA855144 and https://www.biosino.org/node, OEP003571.

## Author contributions

JiwL and X-HZ designed the experiments. JinL and XW analyzed the data and wrote the manuscript. JiaL and XL carried out the laboratory experiments. JiwL, X-HZ, XW, and JinL revised the manuscript. All authors contributed to the article and approved the submitted version.

## Funding

This work was supported by the National Natural Science Foundation of China (nos. U1806211, 92051115, and 41976101), the Fundamental Research Funds for the Central Universities (nos. 202141009 and 202172002), and the National Key Research and Development Program of China (no. 2018YFE0124100). Data and samples were collected onboard of *R/V* Beidou implementing the open research cruise NORC2019-01 supported by the NSFC Shiptime Sharing Project (no. 41849901).

## Conflict of interest

The authors declare that the research was conducted in the absence of any commercial or financial relationships that could be construed as a potential conflict of interest.

## Publisher’s note

All claims expressed in this article are solely those of the authors and do not necessarily represent those of their affiliated organizations, or those of the publisher, the editors and the reviewers. Any product that may be evaluated in this article, or claim that may be made by its manufacturer, is not guaranteed or endorsed by the publisher.

## Footnotes

^1^ http://www.arb-silva.de

^2^ http://sourceforge.net/projects/rdp-classifier

^3^ https://gephi.org
