# Supplementary material for: Comparison of assembly process and co-occurrence pattern between planktonic and benthic microbial communities in the Bohai Sea
Source: Front Microbiol. 2022 Sep 29;13:1003623. doi: 10.3389/fmicb.2022.1003623 (PMC9641972; doi:10.3389/fmicb.2022.1003623)
Supplement: Supplementary file 1 [file Data_Sheet_1.docx]

Supplementary Material

Comparison of assembly process and co-occurrence pattern between planktonic and benthic microbial communities in the Bohai Sea

**Jinmei Liu^1†^, Xiaolei Wang^1†^, Jiao Liu^1^, Xiaoyue Liu^1^, Xiao-Hua Zhang^1,2,3^ and Jiwen Liu****^1,2,3^***

*** Correspondence:** Jiwen Liu: [liujiwen@ouc.edu.cn](mailto:liujiwen@ouc.edu.cn)

## Supplementary Tables

**Supplementary Table 1** Permutational multivariate analysis of variance (PERMANOVA) based on Bray-Curtis distance. Sur_FL, FL community in surface water, Sur_PA, PA community in surface water; Bot_FL, FL community in bottom water; Bot_PA, PA community in bottom water; Sed, sedimentary community

| **Group** | **F Model** | **R^2^** | ***P* value** | ***P* adjusted** |
| --- | --- | --- | --- | --- |
| Sur_FL/Sur_PA | 8.720 | 0.237 | 0.001 | 0.01 |
| Sur_FL/Bot_FL | 9.044 | 0.244 | 0.001 | 0.01 |
| Sur_FL/Bot_PA | 12.932 | 0.316 | 0.001 | 0.01 |
| Sur_FL/Sed | 40.708 | 0.592 | 0.001 | 0.01 |
| Sur_PA/Bot_FL | 18.413 | 0.397 | 0.001 | 0.01 |
| Sur_PA/Bot_PA | 9.976 | 0.263 | 0.001 | 0.01 |
| Sur_PA/Sed | 29.614 | 0.514 | 0.001 | 0.01 |
| Bot_FL/Bot_PA | 14.106 | 0.335 | 0.001 | 0.01 |
| Bot_FL/Sed | 58.552 | 0.676 | 0.001 | 0.01 |
| Bot_PA/Sed | 14.688 | 0.344 | 0.001 | 0.01 |

**Supplementary Table 2** Analysis of similarities (ANOSIM) based on Bray-Curtis distance

| **Group** | **R** | ***P* value** | ***P* adjusted** |
| --- | --- | --- | --- |
| Sur_FL/Sur_PA | 0.617 | 0.001 | 0.001 |
| Sur_FL/Bot_FL | 0.507 | 0.001 | 0.001 |
| Sur_FL/Bot_PA | 0.842 | 0.001 | 0.001 |
| Sur_FL/Sed | 1 | 0.001 | 0.001 |
| Sur_PA/Bot_FL | 0.877 | 0.001 | 0.001 |
| Sur_PA/Bot_PA | 0.701 | 0.001 | 0.001 |
| Sur_PA/Sed | 0.995 | 0.001 | 0.001 |
| Bot_FL/Bot_PA | 0.720 | 0.001 | 0.001 |
| Bot_FL/Sed | 1 | 0.001 | 0.001 |
| Bot_PA/Sed | 0.823 | 0.001 | 0.001 |

**Supplementary** **Table 3** Environmental parameters of the seawater samples

| **Station** | **Pressure (dbar)** | **Temperature (℃)** | **Salinity (PSU)** | **Density (kg/m^3^)** | **Oxygen (mg/L)** | **Fluorescence (mg/m^3^)** | **pH** | **Nitrite (****mg/L)** | **Nitrate (mg/L)** | **Ammonium (mg/L)** | **Phosphate (mg/L)** | **Silicate (mg/L)** |
| --- | --- | --- | --- | --- | --- | --- | --- | --- | --- | --- | --- | --- |
| B1S | 3.04 | 24.68 | 31.84 | 21.05 | 7.95 | 0.54 | 7.954 | ND | 0.003 | 0.010 | 0.017 | 0.176 |
| B1B | 26.10 | 18.53 | 31.92 | 22.78 | 6.77 | 0.66 | 7.674 | 0.006 | 0.013 | 0.024 | 0.016 | 0.194 |
| B3S | 3.16 | 28.37 | 29.72 | 18.31 | 7.55 | 1.74 | 8.265 | ND | 0.018 | 0.014 | 0.011 | 0.111 |
| B3B | 25.23 | 17.95 | 31.97 | 22.96 | 6.92 | 0.56 | 7.544 | 0.002 | 0.021 | 0.028 | 0.016 | 0.209 |
| B5S | 2.94 | 25.79 | 30.67 | 19.83 | 7.99 | 7.82 | 7.914 | 0.0004 | 0.018 | 0.013 | 0.013 | 0.198 |
| B5B | 23.18 | 17.82 | 31.40 | 22.56 | 4.84 | 0.48 | 7.406 | 0.015 | 0.079 | 0.071 | 0.014 | 0.285 |
| B7S | 2.972 | 26.63 | 31.58 | 20.26 | 7.13 | 4.98 | 8.101 | 0.0003 | 0.033 | 0.012 | 0.014 | 0.138 |
| B7B | 19.11 | 21.83 | 31.76 | 21.81 | 5.38 | 0.54 | 7.88 | 0.024 | 0.053 | 0.010 | 0.019 | 0.208 |
| B9S | 3.05 | 27.81 | 31.63 | 19.93 | 6.86 | 7.17 | 8.091 | 0.0002 | 0.009 | 0.012 | 0.014 | 0.079 |
| B9B | 23.18 | 18.26 | 31.88 | 22.82 | 4.80 | 0.28 | 7.544 | 0.036 | 0.073 | 0.011 | 0.021 | 0.226 |
| B11S | 3.08 | 27.63 | 31.71 | 20.04 | 7.15 | 5.63 | 8.044 | 0.0002 | 0.005 | 0.011 | 0.014 | 0.112 |
| B11B | 20.15 | 18.47 | 31.84 | 22.74 | 4.59 | 0.32 | 7.35 | 0.041 | 0.070 | 0.017 | 0.019 | 0.328 |
| B12S | 3.09 | 26.56 | 31.67 | 20.35 | 7.29 | 2.82 | 7.911 | 0.001 | 0.021 | 0.019 | 0.021 | 0.168 |
| B12B | 20.09 | 19.24 | 31.89 | 22.58 | 4.14 | 0.47 | 7.328 | 0.018 | 0.037 | 0.029 | 0.021 | 0.427 |
| B15S | 3.08 | 27.11 | 29.83 | 18.80 | 6.53 | 1.48 | 8.06 | 0.0002 | 0.010 | 0.013 | 0.016 | 0.250 |
| B15B | 21.16 | 24.59 | 31.51 | 20.83 | 4.24 | 0.79 | 7.761 | 0.010 | 0.024 | 0.047 | 0.021 | 0.422 |
| B17S | 3.01 | 27.56 | 30.47 | 19.13 | 6.79 | 1.79 | 8.211 | 0.0004 | 0.006 | 0.013 | 0.017 | 0.220 |
| B17B | 41.30 | 19.87 | 31.81 | 22.37 | 3.78 | 0.56 | 7.503 | 0.044 | 0.066 | 0.014 | 0.026 | 0.415 |
| B19S | 3.019 | 27.44 | 31.00 | 19.57 | 6.98 | 0.98 | 8.14 | 0.0001 | 0.006 | 0.013 | 0.016 | 0.207 |
| B19B | 31.21 | 18.42 | 32.03 | 22.90 | 5.35 | 0.61 | 7.588 | 0.016 | 0.030 | 0.027 | 0.021 | 0.263 |
| B20S | 3.20 | 27.85 | 31.88 | 20.10 | 7.64 | 1.36 | 8.139 | 0.002 | 0.007 | 0.012 | 0.018 | 0.180 |
| B20B | 26.10 | 18.00 | 32.07 | 23.03 | 3.57 | 0.48 | 7.364 | 0.040 | 0.059 | 0.011 | 0.025 | 0.481 |
| B23S | 3.10 | 28.43 | 29.59 | 18.19 | 7.68 | 1.59 | 8.17 | 0.0003 | 0.004 | 0.013 | 0.016 | 0.186 |
| B23B | 24.97 | 21.84 | 32.09 | 22.05 | 5.04 | 0.49 | 7.65 | 0.003 | 0.013 | 0.051 | 0.023 | 0.361 |
| B27S | 3.02 | 26.54 | 26.55 | 16.52 | 7.04 | 2.01 | 7.834 | 0.013 | 0.108 | 0.016 | 0.016 | 0.259 |
| B27B | 15.13 | 20.96 | 30.60 | 21.16 | 3.82 | 0.69 | 7.508 | 0.010 | 0.109 | 0.120 | 0.017 | 0.327 |
| B30S | 3.07 | 28.51 | 27.18 | 16.36 | 8.96 | 2.73 | 8.223 | 0.000 | 0.104 | 0.009 | 0.014 | 0.476 |
| B30B | 17.14 | 21.04 | 31.17 | 21.57 | 3.88 | 0.52 | 7.606 | 0.003 | 0.072 | 0.032 | 0.016 | 0.372 |
| B32S | 3.09 | 23.79 | 31.42 | 21.00 | 7.49 | 1.51 | 7.787 | 0.001 | 0.104 | 0.018 | 0.017 | 0.239 |
| B32B | 15.10 | 23.04 | 31.47 | 21.25 | 7.17 | 1.2 | 7.676 | 0.001 | 0.159 | 0.019 | 0.016 | 0.238 |

ND, not detected

**Supplementary Table 4** Neutral modeling of the relative contribution of stochastic processes to the assembly of different communities

| **Group** | **R^2^** | **m** |
| --- | --- | --- |
| Sur_FL | 0.829 | 0.151 |
| Sur_PA | 0.784 | 0.128 |
| Bot_FL | 0.789 | 0.424 |
| Bot_PA | 0.665 | 0.573 |
| Sed | 0.531 | 0.933 |

**Supplementary Table 5** Comparison of network-level topological features of different communities

|  | Sur_FL | Sur_PA | Bot_FL | Bot_PA | Sed |
| --- | --- | --- | --- | --- | --- |
| **Edges** | 917 | 1033 | 6398 | 18398 | 18337 |
| **Nodes** | 122 | 151 | 279 | 403 | 259 |
| **Clustering Coefficient** | 0.669 | 0.702 | 0.904 | 0.949 | 0.962 |
| **Average Path Length** | 3.089 | 4.297 | 3.521 | 3.347 | 1.387 |
| **Modularity** | 0.248 | 0.091 | 0.058 | 0.044 | 0.012 |
| **Graph Density** | 0.124 | 0.091 | 0.165 | 0.227 | 0.549 |
| **Network Diameter** | 9 | 10 | 11 | 10 | 6 |
| **Average Degree** | 15.033 | 13.682 | 45.864 | 91.305 | 141.599 |
| **Centralization Betweenness** | 0.105 | 0.255 | 0.218 | 0.155 | 0.021 |
| **Centralization Degree** | 0.264 | 0.162 | 0.213 | 0.243 | 0.215 |
| **Positive Nodes** | 99.89% | 98.64% | 96.58% | 97.87% | 99.26% |
| **Negative Nodes** | 0.11% | 1.36% | 3.42% | 2.13% | 0.74% |

## Supplementary Figures


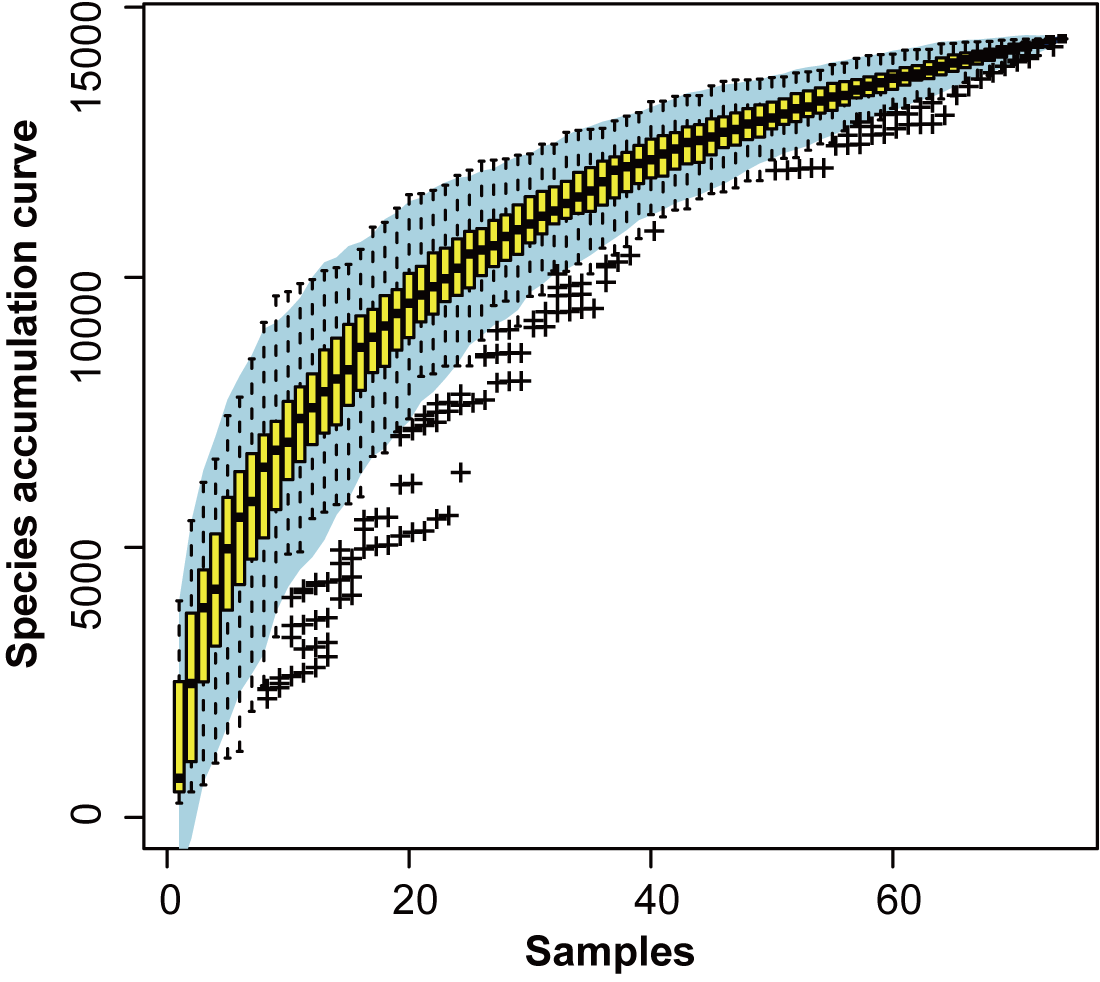


**Supplementary Figure 1** Species accumulation curve based on the OTU level community

**
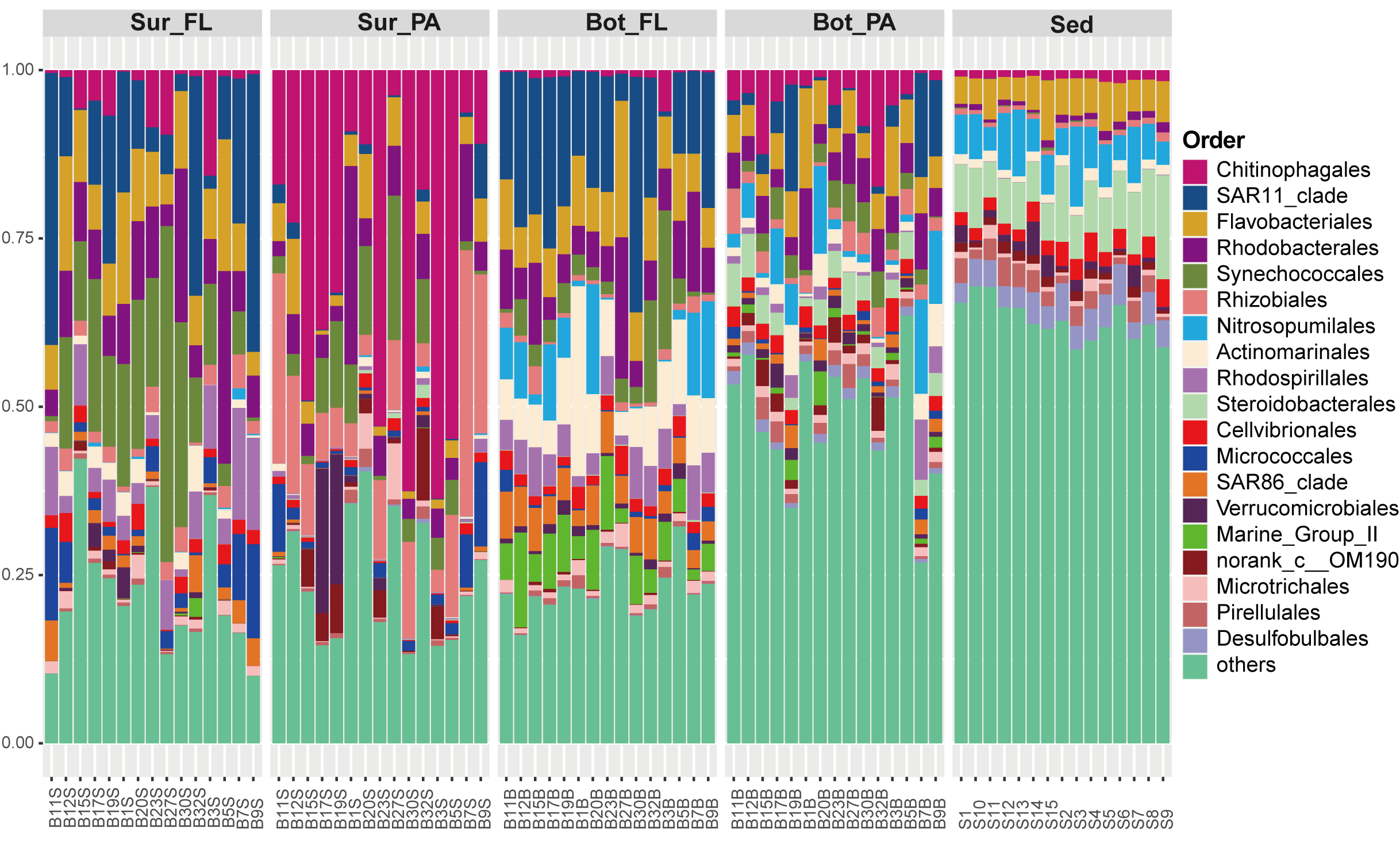
**

**Supplementary Figure 2** Composition of microbial communities at the order level. Sur_FL: FL community in surface water; Sur_PA: PA community in surface water; Bot_FL: FL community in bottom water; Bot_PA: PA community in bottom water; Sed: sedimentary community


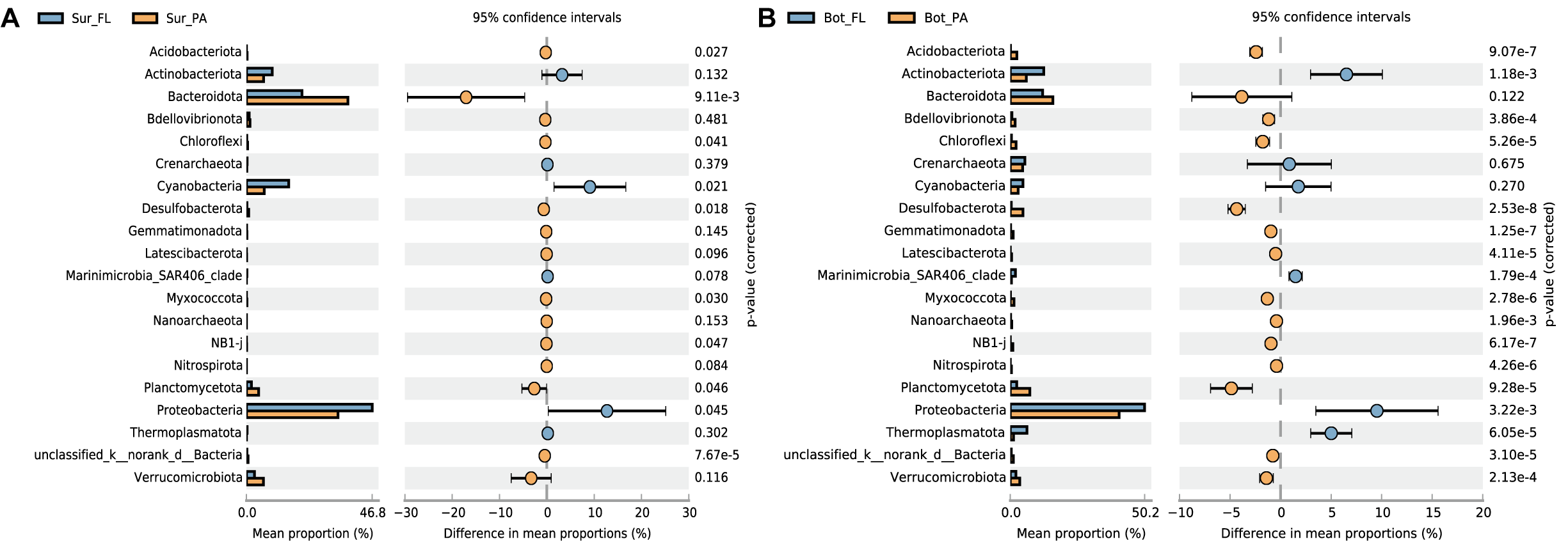


**Supplementary Figure 3** Differences in community composition between lifestyles in surface (A) and bottom (B) water at the phylum level


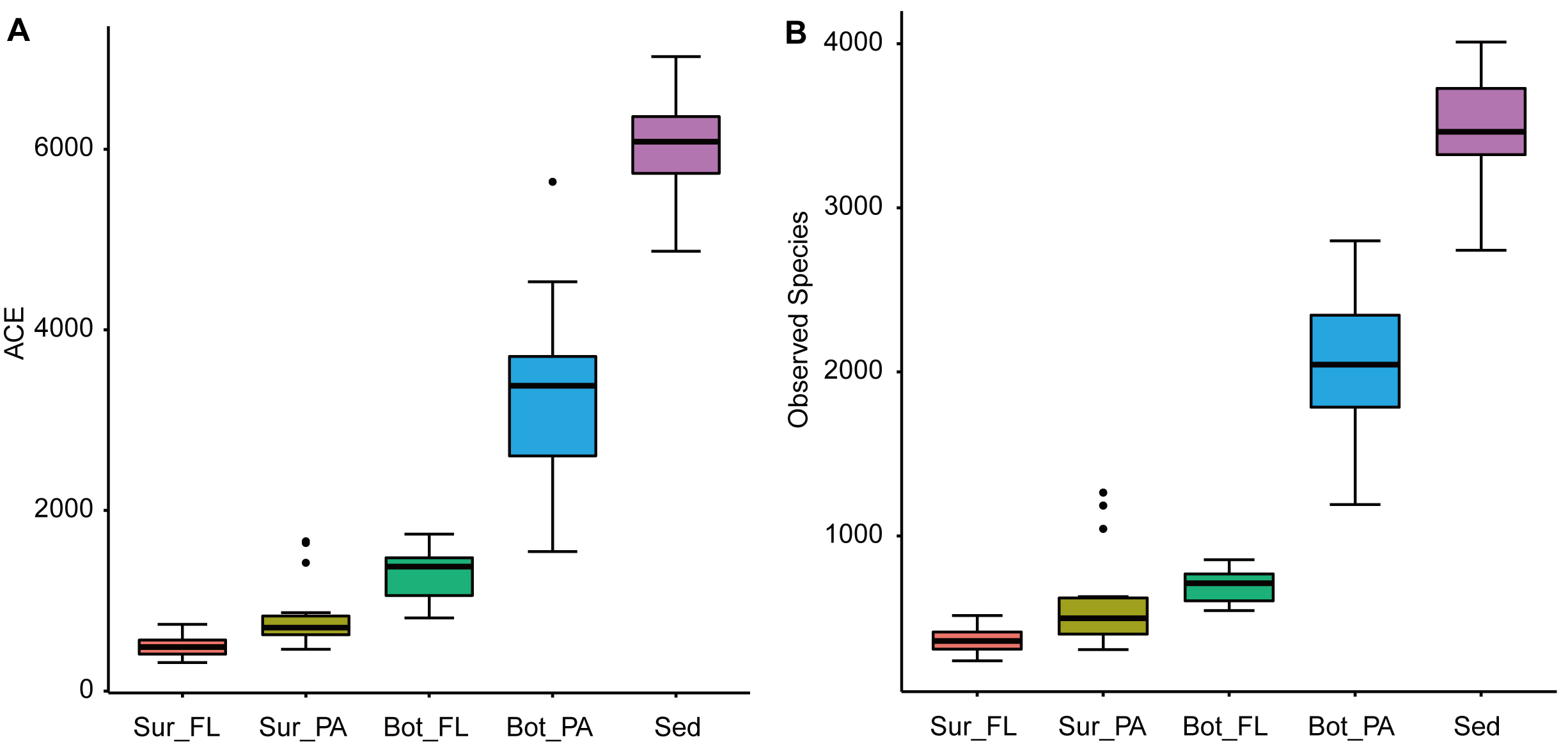


**Supplementary Figure 4** Diversity of microbial communities at the OTU level. (a) ACE index of different communities; (b) Number of observed species of different communities. Sur_FL, FL community in surface water; Sur_PA, PA community in surface water; Bot_FL, FL community in bottom water; Bot_PA, PA community in bottom water; Sed, sedimentary community


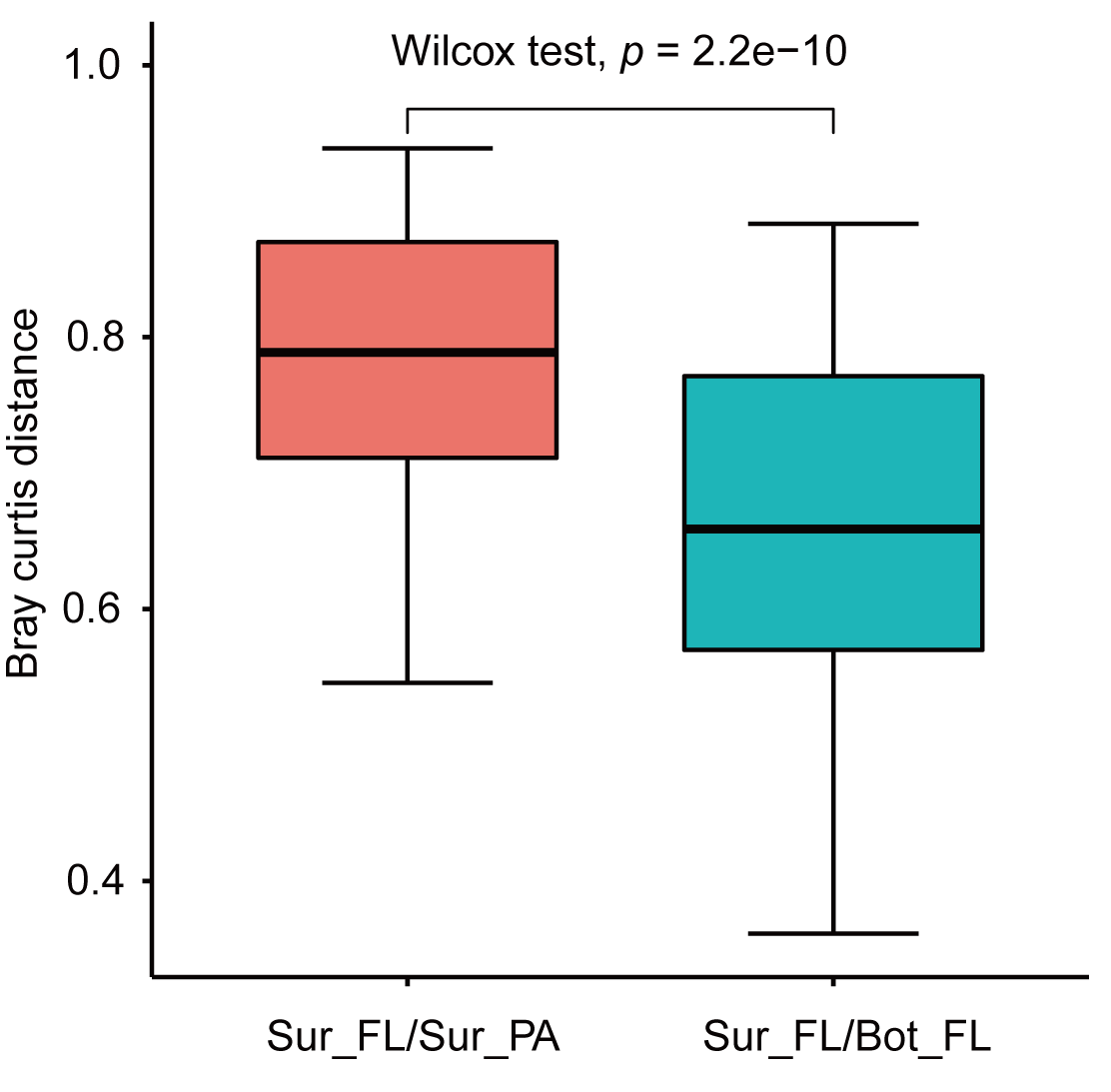


**Supplementary Figure 5** Bray-Curtis distance of Sur_FL/Sur_PA and Sur_FL/Bot_FL communities.
